# Toward building a better scaffold: how types of mentor support inform mentor-mentee match relationship quality

**DOI:** 10.3389/fpsyg.2023.1259040

**Published:** 2024-01-03

**Authors:** Bo Feng, Michael J. Nakkula, Fei Jiang

**Affiliations:** ^1^Center for Ideological and Political Education, Northeast Normal University, Changchun, China; ^2^Human Development and Quantitative Methods Division, University of Pennsylvania Graduate School of Education, Philadelphia, PA, United States

**Keywords:** mentor support, types of purposes, match relationship quality, future outlook, character development

## Abstract

In the field of youth mentoring, mentor support, as an important scaffold for youth development, is linked to match relationship quality between mentors and mentees. This study examined associations between the support provided by different categories of mentors and internal match quality among 240 mentors in youth mentoring programs. Four clusters of mentors emerged, representing different combinations of purposes for mentor-mentee interactions. Mentors who focused less on the character development of their mentees showed reduced benefits in other forms of interactions, such as fun, sharing, future outlook, or academics in promoting high overall mentor-mentee internal match quality, including relational quality and instrumental quality. While mentors who focused less on future outlook in their mentoring interactions showed reduced benefits for other purposes in promoting internal instrumental quality. These findings were not influenced by mentors’ demographic differences such as gender, age, race, and educational background. The significance of the findings for future research and practice is discussed.

## 1 Introduction

Mentoring as an important life asset in the lives of young people holds great promise in the field of youth development. Mentor support has been associated with positive outcomes in areas such as academics, peer and family relationships, self-esteem and reduced risk-taking behavior (Parra et al., 2002; Cavell et al., 2009; Herrera et al., 2011, 2013). There are many programs in the United States that engage in youth development, such as 4-H, Boys and Girls Clubs, Scouting, Best Kids (BK), Connecting Generations (CG), Big Brothers Big Sisters (BBBS), YMCA, Girl POWER!, and smaller local organizations. Mentoring is commonly divided into formal and natural (Chao et al., 1992) mentoring approaches according to whether or not there is organized recruitment, support, and supervision. In the case of formal mentoring, a mentor is recruited, trained by the project and matched with a mentee to engage in various activities and address developmental growth and potentially risky behaviors (Lakshminarayanan et al., 2022). Over the past few decades, formal mentoring programs have attracted large numbers of youth and mentors to participate in programs in their schools and communities, and millions of youth have benefited from them. The benefits of youth mentoring are widely viewed as the result of caring mentor-mentee relationships. In fact, mentoring relationship quality has been found to be an important moderator of the benefits youth achieve from mentoring (Rhodes, 2005). High-quality mentoring relationships could exert a positive impact on adolescent development in the emotional, social, and academic domains (DuBois et al., 2011; Bayer et al., 2015; Silke et al., 2019). Conversely, if such bonds are not formed, mentees and mentors may disengage from the match before the match relationship lasts long enough to have a positive impact (Herrera et al., 2000). The purpose of this study is to understand the relationship between the support provided by different categories of mentor purposes and match relationship quality, with the aim of providing better scaffolding for youth. The existing literature on mentor support shows a basic understanding of the roles mentors play and the types of support that influences match relationship quality. However, there is still much space for further exploration, such as the need for additional understanding of the connections between types of mentor support and the impact on mentoring relationship quality.

### 1.1 Mentor support and mentor purpose

People may experience different types of mentoring at different stages of their lives, which could exert significant influences on their abilities, life goals, values, etc. As a critical period for individuals to seek and explore the meaning of life (Devogler and Ebersole, 1983), youth often require external supports to guide them and help them continuously shape the course of their lives and improve their abilities. As an important factor in guiding adolescents toward positive outcomes, mentors can engage in mentoring to increase desirable (e.g., academic performance, job performance) behavior and decrease undesirable (e.g., school drop-out, substance use) behavior (Eby et al., 2008), assuming the responsibility for developing the mentees’ competence and character. In a broader sense, mentor support can be divided into the support given to mentors from external sources (such as programs, parents, etc.) and the support that mentors provide to their mentees. As a necessary scaffold (Vygotsky, 1978), we define mentor support in this study as mentors providing ongoing structures and interactions to mentees in mentor-mentee activities, such as building relationships, introducing opportunities, and developing abilities. This scaffold is intended to enable youth to reach and consolidate a higher level of competence and performance than would be received without such support.

The specific type of support provided by mentors depends on their purpose in the mentoring activities. Mentor purpose is defined as what mentors want to do or accomplish in the match (Nakkula and Harris, 2014). Specific mentoring activities and conversation topics are intended to align with the mentor’s purposes for engaging in the match and supporting their mentees accordingly. Mentors provide various types of support for different types of mentees. Kram (1985) identified two types of mentor support for adults in organizational settings: career-related and psychological support. Follow-up work had empirically supported Kram’s classification. Noe (1988) developed a measure confirming that career-related and psychosocial support are two unique mentor functions for various categories of mentees. The purpose of mentors, which is reflected in their motivation for mentoring (i.e., Allen, 2003), is closely related to the support they provide. For example, when mentors focus on self-improvement, specifically for older mentees, they are more inclined to provide support for career development. On the other hand, when their purpose is to benefit others and seek intrinsic satisfaction, they tend to provide more socio-psychological support (Allen, 2003).

In addition, some scholars have classified mentor support into three categories: emotional support, instrumental support, and companionship (Cohen and Wills, 1985). Instrumental support generally connotes the provision of concrete assistance in some way, such as teaching new skills or providing material assistance. Emotional support focuses on helping mentees feel understood (experience empathy) and share and process their feelings; these processes are also believed to enhance perspective-taking skills and promote empathy. Contrary to emotional and instrumental support, which is designed to address emotional distress or seek help in solving problems, companionship is a purely enjoyable interaction, such as sharing leisure activities, trading life stories, and sharing humorous anecdotes (Rook, 1995); the purpose of this approach is to help counter mentee isolation and loneliness by providing social engagement. For some youth, companionship is unique in that it is a welcome respite provided by mentors in times of distress (Morrow and Styles, 1995), and it also contributes to positive adolescent outcomes. Nakkula and Harris (2010, 2014) viewed companionship as a form of social engagement that is critical to emotional support. Overall, Nakkula and Harris conceive of mentor support in two broad categories: relational and instrumental support. Within this framework, they view mentor purposes as falling within the instrumental area, including future orientation, character development, and academic or related skill development, while relational purposes would include providing empathy, care, social connectedness, and simply playful or recreational interactions. Instrumental purposes aim to assist youth in making constructive life decisions for themselves and potentially to help others and society more broadly, while relational purposes are rooted in the goals of supporting mentees’ social and emotional development without necessarily focusing on specific instrumental or achievement-oriented outcomes. A distinction between “helping self and others” and “social connection and enjoyment” lies between these two purposes. To further explore the connection between purposes and support, Nakkula and Harris (2010) pointed out that mentors who focus on traditional purposes such as character development, future outlook development, or academics purpose typically provide instrumental support to their mentees (e.g., giving advice to mentees, doing homework together). In contrast, mentors who focus on relational purposes like fun or sharing purposes are more inclined to provide emotional support.

### 1.2 Relationship quality

Mentoring as an intervention aims to pair children with older youth or adults to foster a meaningful relationship with a non-parental figure (DuBois et al., 2011). Previous studies have found that the higher the level of intimacy reported in the mentoring relationship, the better the outcome predicted (DuBois and Silverthorn, 2005). It is worth pointing out that too much intimacy may not always be beneficial for the relationship (Dutton et al., 2023). Higher quality mentoring is associated with youth having greater self-esteem, fewer alcohol problems, and fewer depressive symptoms (Whitney et al., 2011). Simultaneously, it has been found that the presence of a strong emotional connection is a distinguishing feature of those mentoring relationships that are associated with better outcomes, such as improvements in perceptions of scholastic competence, feelings of self-worth as well as improvements in social and emotional functioning (Sánchez et al., 2006; Spilt et al., 2012; Cavell and Elledge, 2013; Kanchewa et al., 2016). Thus, it is reasonable to expect that mentoring relationship quality predicts youth progress and that higher relationship quality is associated with better outcomes.

Match relationship quality refers to the evaluation of the overall state of the mentoring relationship, which is conceptualized as an affective assessment of the overall state of the relationship that ranges from low to high quality and is distinct from mentor and mentee role behaviors (Eby et al., 2010). Nakkula and Harris (2014) define match quality as the characteristics of relationships between adults and youth that are specific to the mentoring experience and are thought to influence the mentees’ outcomes (growth and development). From a relational mentoring perspective, mentoring relationships are dynamic because they vary in quality over time based on mentors’ and mentees’ experiences and interactions with each other throughout the match (Ragins, 2012).

The existing research also indicates that the factors affecting match relationship quality are mainly divided into subjective and objective factors. Objective factors that have been found to affect match quality include mentors’ interpersonal knowledge, skills, and abilities, such as the mentor’s ability to demonstrate empathy (Pryce, 2012; Spencer and Basualdo-Delmonico, 2014; Doty et al., 2019); the mentees’ family environment, hobbies, and other personal characteristics (Britner and Kraimer-Rickaby, 2005; Raposa et al., 2016) and the differences in age, gender, and race between the mentors and mentees (Sanchez et al., 2013; Albright et al., 2017; Raposa et al., 2017). Subjective factors in previous studies include the perceived impact of mentors’ personality, self-efficacy, and motivation (Karcher et al., 2005, 2010; Keller and Pryce, 2010; Leyton-Armakan et al., 2012; Martin and Sifers, 2012; Raposa et al., 2016) as well as the mentees’ psychological state (Gormley, 2008) and differences in needs and perceptions between the mentors and mentees (Karcher et al., 2005; Raposa et al., 2019).

Various indicators have been used to measure match relationship quality. From the perspective of the interpersonal relationship investment model, relationship quality is judged based on a weighing of costs and benefits (Tran et al., 2019; Drew et al., 2020). Costs refer to the inputs in the mentoring, such as emotional support, instrumental support, and time commitments, while benefits refer to the development that adolescents gain from the mentoring relationship. According to Feldman (1999), mentorships are low-quality or dysfunctional, if they fail to meet the needs of either person or if long-term costs outweigh long-term benefits. Relationship quality has also been discussed in terms of satisfaction with the relationship, mutuality of benefits, and relational depth (Huston and Burgess, 1979). Some studies have found that due to the relational nature of youth mentoring, relationship quality is often conceptualized and measured using constructs that tap into the bond between mentor and mentee. For example, closeness (Bayer et al., 2015), dependency (Goldner and Mayseless, 2009), relationship satisfaction (Leyton-Armakan et al., 2012), warmth and trust (Farruggia et al., 2013) are all measures based on the research above. Nakkula and Harris (2005) determined that internal match quality, external match quality and match structure are critical indicators of overall match quality. Internal match quality reflects how participants feel about what is done in the match and about their relationship with each other; external match quality refers to the outside nature of support for the match, including family and program support; and structure refers to the ways in which discussion and activities are carried out in the match.

High relationship quality is an important element of an effective mentoring program. Relationship quality has been identified as an indicator of mentoring effectiveness given its association with a range of outcomes (e.g., Allen and Eby, 2003; Allen et al., 2006; Poteat et al., 2009). Nakkula and Harris (2005) argued that because of the importance of match relationship quality to a range of outcomes, it should be better understood in order to provide “best practice” guidance for both mentors and mentees. A growing body of research is deepening our understanding of relationship quality to the mentoring process (e.g., DuBois and Silverthorn, 2005; Rhodes and DuBois, 2006; Whitney et al., 2011; Chan et al., 2013). The development of adolescents in the domains of social–emotional, cognitive, and identity presupposes the development of a close, caring relationship, such as those that can occur through high quality mentoring (Rhodes et al., 2006). If a bond is not formed, mentors and mentees may disband before the relationship has an effect. For most mentoring programs, higher quality relationships produce better outcomes and may be the active ingredient by which mentoring effects are realized (Rhodes et al., 2006; Herrera et al., 2007; Kanchewa et al., 2016).

### 1.3 The link between mentor purpose and match relationship quality

Mentor purpose and match relationship quality share a strong, intuitive, and nuanced connection (Nakkula and Harris, 2010). From an instrumental perspective, a high-quality mentoring relationship is marked by the mentees’ perception that they are supported in pursuing a specific goal or completing an important task and the mentors’ perception that they are helpful in supporting their mentees’ efforts to achieve their goals (Nakkula and Harris, 2005). For example, when mentors structure their matches around academic support and it is perceived as helpful by their mentees, instrumental relationship quality tends to be high. Similarly, from a relational perspective, match quality tends to be high when mentors provide emotional support that meets mentees’ needs in this area. Specifically, both fun and sharing purposes are broad predictors of internal relational quality (Nakkula and Harris, 2005).

Nakkula and Harris (2010) concluded that the mentors’ purpose in structuring the match informed the nature of match quality. However, in the data they reported, future outlook and character development purpose, had little positive or negative effect on relationship quality, which seems inconsistent with the expected link between the purpose of the mentors and match quality. We believe that mentors are highly motivated and can provide different types of support depending on their purpose. In the actual mentoring process, mentors often enter their matches with the desire to help their mentees with behavioral issues that have been troubling, which affects the mentoring relationship. But in their study, Nakkula and Harris (2010) did not find strong evidence for character development purpose being associated with instrumental match quality. Based on their findings, they concluded that match quality, including instrumental match quality, was relatively high when instrumental purposes such as character development were moderated by a focus on “sharing” or the exchange of personal information in the match.

This study attempts to take a different approach by clustering the mentors based on their actual interactions. We focus on the initiative of the mentor, believing that mentors determine their purpose in mentoring activities according to their own preferences and the characteristics of their mentees, and then provide support consistent with the purpose. We are not exploring the priority order of different purpose combinations in mentor-mentee match quality. Instead, we are identifying the significant roles of different purposes or different combinations of purposes through an internal correlation analysis between different clusters of mentors organized by purpose. We aim to understand the link between different categories of purpose-based mentor support and match quality.

### 1.4 Research questions

Based on the foregoing, we hypothesize that mentors would cluster into different groups based on their patterns of focus during mentoring. We expect that focus composites that combine instrumental and relational aspects will have an impact on match relationship quality, but we are exploring rather than predicting the specific impact. Whether mentors would vary in their preference of focus during mentoring activities based on their demographic characteristics remains an open question, so we are exploring this possibility to better understand the potential factors that may exert influences on mentors’ purposes and the overall match relationship quality. Thus, this study addressed the following questions:

1. Can mentors be clustered based on their different combinations of purposes in mentoring?
2. Does match quality vary on a cluster basis? If mentors can be clustered based on the different combinations of purposes in mentoring, what are the relationships between the different purposes on which mentors focus and the match quality within each cluster?
3. Are there significant demographic differences (mentors’ gender, race, age, and education background) among the clusters?

## 2 Materials and methods

### 2.1 Participants and procedure

This study analyzed survey data collected from two school-based mentoring programs in 2017 and 2019. Both programs aim to provide mentoring to support children in becoming emotionally strong, resilient, and socially competent individuals who can successfully navigate school and life. The pairing of mentors and mentees was determined by an outside mentoring program that supervised the formal mentoring relationships.

The data used in this study were drawn from a larger research project studying mentor purposes in interactions, mentor-mentee match relationship quality, and external support. Participants (*N* = 240) in this research were mentors involved in the larger study. Mentors ranged in age from 14 to 86 years old (*M* = 40.04, SD = 16.352). 151 were female (62.92%) and 89 were male (37.08%).

The mentors identified as 47.08% White Americans (*n* = 113), 18.75% Black (*n* = 45), 7.50% Asian (*n* = 18), 5.42% Hispanic (*n* = 13), and 21.25% reported being other racial backgrounds (*n* = 51). Among the mentors who participated in the survey, the majority reported the highest level of education to be a bachelor’s degree or higher (86 bachelor’s degree, 35.83%; 67 graduate degree, 27.92%), 14.58% gained associate’s degrees or vocational certificates (*n* = 35), 7.08% were in some college (*n* = 17), 2.50% graduated in high school (*n* = 6), and 12.08% were in some high school (*n* = 29).

### 2.2 Measures

This article follows the framework of *match relationship quality* presented by Nakkula and Harris (2005) by studying the characteristics of relationships between adults and youth that are specific to the mentoring experience and thought to influence the mentee’s outcomes. Sections of the *Match Characteristics Questionnaire*, a comprehensive survey of mentors’ approaches to and experiences of their matches (Harris and Nakkula, 2018) were used in this research, including *match structure scales* (mentors’ priorities for match activities and level of attention to purposes relating to fun, sharing, character development, future outlook, and academics) and Internal quality scales (mentors’ experiences of varying mentor-mentee relational and instrumental aspects of the match). Specific information on the questionnaire is shown in Table 1.

**Table 1:** Specific information for match characteristics questionnaire revised in 2018.

Scales	Subscales and specific indexes
Internal quality scales	Internal relational match quality subscale
	Compatibility
	Handle issues
	Closeness
	Discomfort
	Satisfaction
	Competence
	Internal instrumental match quality subscale
	Nonacademic support-seeking
	Academic support-seeking
Match structure scales	Fun purpose subscale
	Sharing purpose subscale
	Character development purpose subscale
	Future outlook purpose subscale
	Academics purpose subscale
External quality scales	Program support subscale
	Parent engagement subscale
	Friend/family support subscale
	Interference subscale

#### 2.2.1 Match structure scales

These scales examine mentors’ purposes from five types of interactions, including fun, sharing, character development, future outlook, and academics, which measure distinct spaces along a playful-to-conventional continuum. Among them, fun and sharing constitute the relational structure, while character development, future outlook, and academics together build up the instrumental structure. The scales altogether consist of 20 items, with each type of purpose incorporating 4 items. All items are rated by a 6-point Likert scale (1 = not important, 6 = most important). Higher ratings imply higher levels of attention to the corresponding purpose in mentoring.

##### 2.2.1.1 Fun purpose subscale

The fun purpose reflects the most purely playful orientation (Nakkula and Harris, 2010) and demonstrates mentors’ regard for spending time with their mentees in interesting and enjoyable activities. This purpose aims, in part, to enhancing the connection between mentors and mentees through engagement in enjoyable and low-stress activities. The fun purpose subscale (*α* = 0.77) involves 4 items: “having times when you do nothing but fun things with your mentee,” “making time to goof around, laugh, and have light-hearted fun with your mentee,” “having time when you and your mentee just hang out together (no particular activity to do),” and “having fun (yourself) while you are with your mentee.”

##### 2.2.1.2 Sharing purpose subscale

The sharing purpose demonstrates how much the mentors value talking with their mentees and sharing their own experiences (Nakkula and Harris, 2010). It refers to the process of reducing unfamiliarity and distance in the mentoring process through a series of meaningful conversations. The sharing purpose subscale (*α* = 0.68) measures social and emotional interactions and support within the match. It includes 4 items: “encouraging your mentee to talk about whatever he/she wants to talk about (even unproductive stuff),” “focusing on feelings and emotional things with your mentee,” “asking your mentee about the things he/she enjoys when you are not together,” and “spending time just talking with your mentee.”

##### 2.2.1.3 Future outlook purpose subscale

The future outlook purpose involves goal setting and future orientation along the lines of adult conventionality, such as exposing the mentees to new ideas and experiences or helping them develop skills and interests (Nakkula and Harris, 2010). In other words, the future outlook purpose is to demonstrate mentors’ value for activities related to mentees’ future planning and enable them to assist mentees in setting life goals and planning for their paths ahead. This helps to organize and motivate the mentees to adjust their attitudes, manage their behaviors, and move forward toward their desired future. The subscale (*α* = 0.78) primarily measures mentors’ valuation of interactions that involve goal setting and future orientation. While it was not the single strongest predictor of any quality indicator, it is a broadly useful predictor of relationship quality. The future outlook purpose subscale can explain a variance across the relationality-instrumentality continuum. The subscale includes 4 items: “encouraging your mentee to push beyond what is comfortable or easy (to expect more of him/herself),” “exposing your mentee to new ideas and experiences,” “getting your mentee to develop stronger skills and interests,” and “getting your mentee to think about serious issues in his/her life (school, relationships, etc.).”

##### 2.2.1.4 Character development purpose subscale

The character development purpose indicates the degree to which mentors value interactions that improve the mentees’ character (e.g., being honest, responsible or kind to others) (Nakkula and Harris, 2010). The character development purpose is for mentors to assist their mentees in comprehending, valuing, and embodying essential moral values. The character development purpose subscale (*α* = 0.76) reflects a substantially conventional purpose and essentially evaluates how much mentors value activities focused on mentees’ maturation and psychosocial development. The character development purpose subscale includes 4 items: “getting your mentee to develop his/her character (be honest, responsible, etc.),” “teaching your mentee to manage or improve his/her behavior (control impulses, make better decisions, etc.),” “getting your mentee to care more about other people,” and “teaching your mentee social skills (like table manners, how to meet people, etc.).”

##### 2.2.1.5 Academics purpose subscale

The academics purpose measures how much mentors prioritize interactions devoted to improving their mentees’ academic performance such as improving attitude toward school or helping with schoolwork (Nakkula and Harris, 2010). To be specific, it means for mentors to assist their mentees in acquiring and mastering various skills and knowledge, with the aim of improving their academic and professional achievements. The academics purpose subscale (*α* = 0.79) reflects an extremely conventional purpose and measures how much mentors prioritize interactions devoted to improving their mentees’ academic performance. The academics purpose subscale includes 4 items: “doing activities with your mentee that get him/her to think (like reading, puzzles, educational games, etc.),” “doing or saying things to improve your mentee’s attitude toward school (or keep it positive if it is already good),” “helping your mentee with schoolwork,” and “involving academics in the mentoring relationship.”

#### 2.2.2 Internal quality scales

In terms of relationship quality, the questionnaire focuses on internal and external quality, reflecting mentors’ positive and negative perspectives on them. Internal quality scales focus on measuring the dynamics directly impacted by matched mentors and youth, which usually include relationship satisfaction, activity satisfaction, and the availability of support. External quality scales measure factors that are not directly influenced by the pair, primarily covering program, parent, peer, and other external influences. For the current study, we explored the relationship between the support from different categories of mentors and internal match quality that occurs within mentor-mentee interactions. The reason we do not consider the external quality scale is that it measures the match relational quality influenced by non-direct participants (mentors and mentees) in the mentoring interactions. Instead, we are directly focused on internal quality scales that reflect the way participants feel about what is done in the match and about each other.

##### 2.2.2.1 Internal relational match quality subscale

The internal relational match quality subscale incorporates 25 items (*α* = 0.92), including items from the compatibility, handle issues, closeness, not distant, satisfaction, and competence subscales. The internal relational match quality scale refers to indications of how a mentor and mentee feel about each other and the way they relate. Compatibility, handle issues, and competence are measured by a 6-point Likert scale as well (1 = completely disagree, 6 = completely agree). These items include, “my mentee and I hit it off right away,” “it is hard for me to deal with my mentee’s behavior,” “I am a good role model for my mentee,” and so on. Closeness, non-distancing, and satisfaction are measured by a 6-point Likert scale (1 = never, 6 = always). These items include, “I feel like my mentee and I are good friends (buddies),” “I feel distant from my mentee,” “I feel like the mentoring relationship is getting stronger,” and so on. Higher ratings imply higher internal relational match quality in mentoring.

##### 2.2.2.2 Internal instrumental match quality subscale

The internal instrumental match quality subscale measures the degree to which mentees are available to various types of mentor support. Internal instrumental match quality subscale incorporates 7 items (*α* = 0.84), includes the nonacademic support-seeking and academic support-seeking items. It is measured by a 6-point Likert scale (1 = never to, 6 = always). These 7 items including, “my mentee is open with me (shares thoughts and feelings),”“my mentee asks for my opinion or advice,” “my mentee makes me aware of his/her problems or concerns,” “My mentee is open with me about his/her friends,” “my mentee talks to me about it when he/she has problems with friends or peers,” “my mentee asks me for help when he/she has difficult schoolwork or a major project to do,” and “my mentee seems to want my help with his/her academics.” Higher ratings imply higher internal instrumental match quality in mentoring.

### 2.3 Analysis

SPSS 26.0 was used for all statistical analysis. After data cleaning and preparation, cluster analysis with hierarchical clustering algorithm was utilized first followed by Hierarchical Clustering Dendrogram together with multiple ANOVAs among different solutions in order to decide the optimal clustering solution among mentors based on their responses to match structure scales. Multiple correlations were then applied in the data analysis process to examine the characteristics of mentor-mentee relationship quality based on clusters.

For question 1, regarding clustering of mentors based on the different combinations of purposes in mentoring using match structure scales, we ran a Hierarchical Cluster Analysis by entering match structure component scores for each mentor. Q-mode HCA for cases brought together samples with common characteristics. HCA was conducted on the calculation method of distance using Squared Euclidean distance between cases and using Between-groups Linkage among clusters. In addition, the method of “Z scores” was selected for individual cases in the standardized processing. In order to determine the best solution, we use the Hierarchical Clustering Dendrogram to choose which solutions are appropriate, and then use ANOVAs to decide the best solution.

For question 2, we first ran ANOVAs to examine the inter-cluster differences of match quality. Then, a correlation analysis was conducted based on the clustering solution from HCA in order to understand the relationship between mentor purpose and match quality intra-cluster. In previous research, Nakkula and Harris (2010) conducted a correlation analysis between a series of purposes and internal match quality, using the whole sample without clustering based on the combination of different purposes that mentors focused on. The results showed that most of the correlation coefficients were low, with only a small portion of them reaching moderate levels. Therefore, after applying the Hierarchical Cluster Analysis, we still chose correlation analysis to further test the relationships between the different purposes which mentors focus on and the match quality within each cluster. To be specific, by comparing correlation coefficients and significance level between the mentors’ focus on different purposes and match quality across the clusters, we attempt to figure out which combination of purposes could predict better outcomes on internal relational quality or internal instrumental quality.

Finally, for question 3, frequencies and means were calculated for each cluster on selected demographic variables (mentors’ age, gender, race, and educational background) and Chi Square tests of independence and ANOVAs with follow-up LSD multiple comparison tests were used to examine cluster differences by these variables. The results are reported next.

## 3 Results

Our findings provide clear evidence that mentors can cluster according to the different combinations of purposes in mentoring. We also found that internal match quality does vary on a cluster basis. In particular, we discovered that mentors who focus less on the character development of their mentees may hinder their efforts in other forms of interactions such as fun, sharing, future outlook or academics in promoting high overall mentor-mentee internal match quality, including relational quality and instrumental quality. Similarly, mentors who focused less on future outlook in their mentoring interactions showed a reduced impact of other purposes in promoting internal instrumental quality. These findings are not influenced by mentors’ demographic differences such as gender, age, race, and educational background. The following sections articulate the research results in more detail and report the specific process.

### 3.1 Mentor clusters

**Figure 1:**
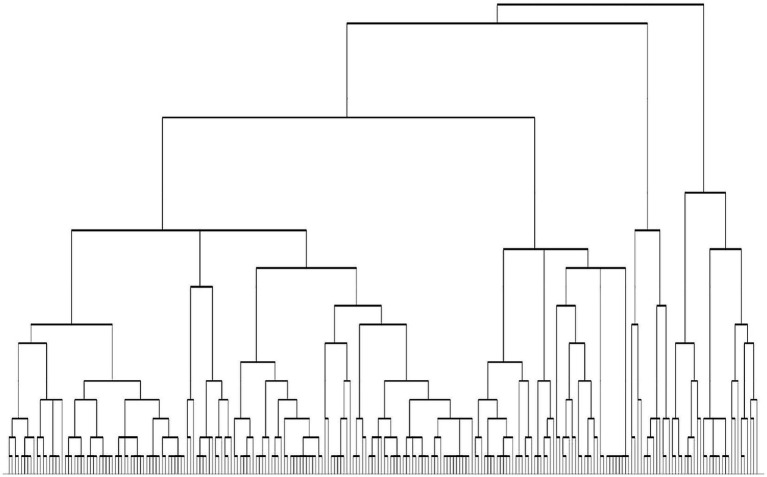
Hierarchical clustering dendrogram.

For the first research question, we found that mentors can be clustered based on the different combinations of purposes they hold, and dividing them into four clusters is the relatively optimal solution. Hierarchical Clustering Dendrogram (Figure 1) implies *K* = 3 and 4 are appropriate solutions for our data. We then applied ANOVAs to decide the best solution. A series of ANOVA tests found the 4-cluster solution possesses lower p levels on all variables than the 3-cluster solution [3-cluster solution: fun *F* (2, 237) = 0.908, *p* = 0.405; sharing *F* (2, 237) = 5.379, *p* = 0.005; character development *F* (2, 237) = 8.780, *p* = 0.000; future outlook *F* (2, 237) = 3.030, *p* = 0.050; academics *F* (2, 237) = 12.939, p = 0.000; 4-cluster solution: fun *F* (3, 236) = 1.472, *p* = 0.233; sharing *F* (3, 236) = 4.358, p = 0.005; character development *F* (3, 236) = 11.599, *p* = 0.000; future outlook *F* (3, 236) = 5.044, *p* = 0.002; academics *F* (3, 236) = 32.300, *p* = 0.000]. Thus, we adopted the 4-cluster solution for our sample.

**Figure 2:**
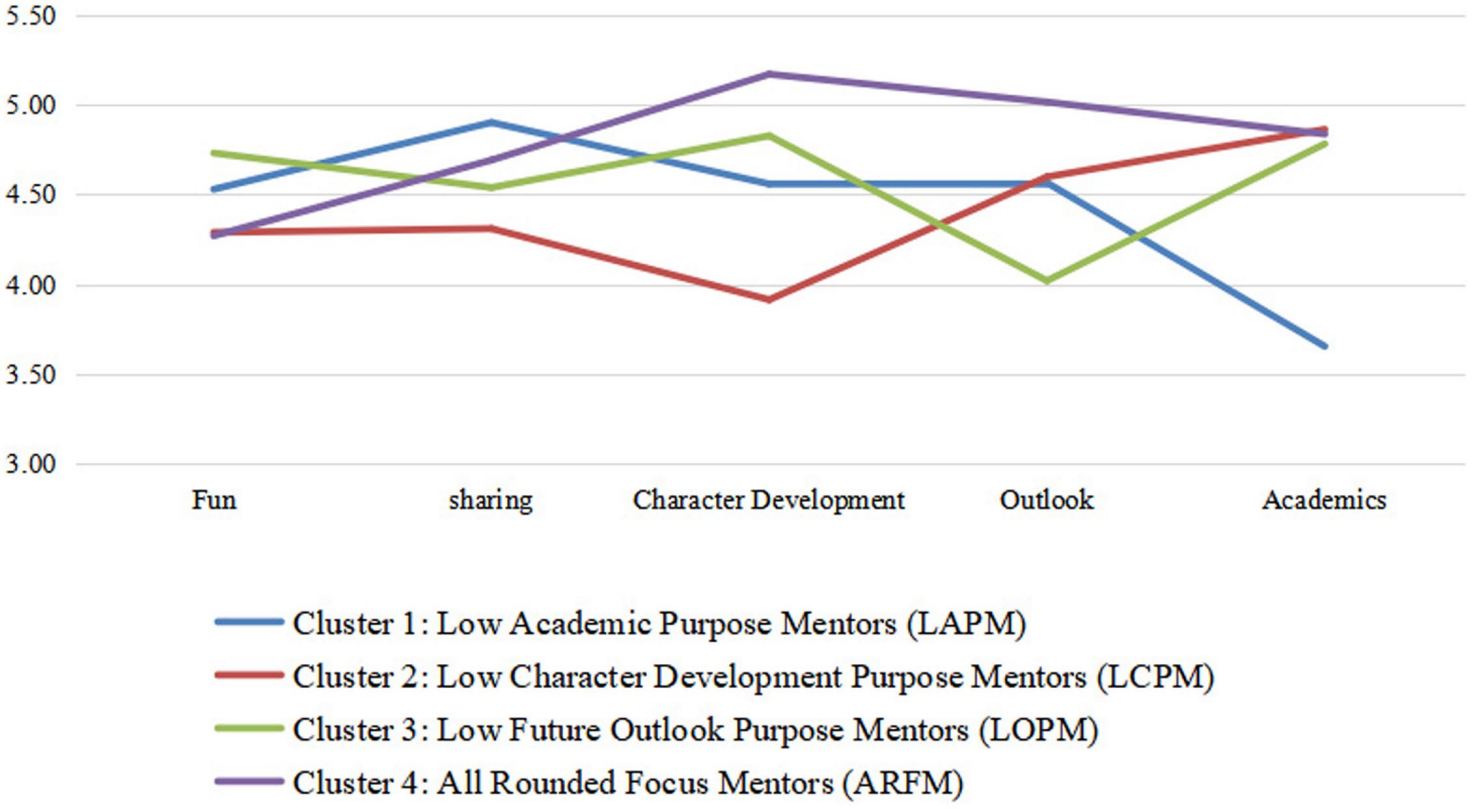
Mean values at levels of attention on different purposes by cluster.

Table 2 lists the mean values of each cluster at levels of attention on different purposes in match structure scales. Before describing each cluster, it should be noted that the fun purpose component exhibits no statistically significant differences among clusters by all solutions. Although multiple comparison tests show statistically significant different between-cluster means on the sharing purpose component, the differences on means for every cluster on this variable were minor (smallest range difference among all purposes except for fun purpose), with ratings ranging from 4.31 and 4.90 (4, very important; 5, extremely important), which reveals close levels of focus on this purpose. We therefore did not take into account the cluster differences in the fun and sharing purpose in our analysis procedures. It is not surprising that the four types of mentors rate comparatively high and cannot be separated in the fun and sharing purposes, as mentors are more or less focused on relational purposes in mentoring (For example, using ice-breaking activities to get to know each other). It should also be mentioned that cluster descriptions are useful in relative terms. Next, we describe each cluster. One of the clusters (cluster 4) has mentor members who rate relatively high importance on each purpose, whereas mentors in the other three clusters (cluster 1, cluster 2, and cluster 3) show relative combinations of purposes that are more biased toward several purposes, and less emphasis on one certain purpose. In other words, although our clustering results achieved the objective of dividing mentors into clusters based on different combinations of purposes, the way it presented is that the mentors lack support for a certain purpose. Figure 2 can more intuitively reflect the mean difference of each cluster. As observed in Figure 2, the four clusters of mentors did not show features of focusing on different combinations of purposes. Instead, three of the clusters exhibited low values for a certain purpose.

**Table 2:** Mean values at levels of attention on different purposes by cluster.

Component	Cluster 1 (*N* = 149): Low academics purpose mentors (LAPM)	Cluster 2 (*N* = 28): Low Character development purpose mentors (LCPM)	Cluster 3 (*N* = 13): Low future outlook purpose mentors (LOPM)	Cluster 4 (*N* = 50): All rounded focus mentors (ARFM)
Fun	4.53	4.29	4.73	4.27
Sharing	4.90	4.31	4.54	4.69
Character development	4.56	3.91	4.83	5.17
Future outlook	4.57	4.60	4.02	5.02
Academics	3.65	4.86	4.78	4.84

Cluster 1, Low Academics Purpose Mentors (LAPM); Cluster 2, Low Character Development Purpose Mentors (LCPM); Cluster 3, Low Future Outlook Purpose Mentors (LOPM); Cluster 4, All Rounded Focus Mentors (ARFM).

#### 3.1.1 High-focus-on-all-purposes cluster

##### 3.1.1.1 Cluster 4: all rounded focus mentors

Mentors in this cluster demonstrated higher levels on all five purposes of mentors-mentees interactions. We labeled this cluster as all rounded focus mentors. Besides fun and sharing, mean purpose ratings on the remaining three components of character development, future outlook, and academics purpose were all around 5 (extremely important; M = 4.84–5.17). These means indicate a group of mentors who attach high importance on all the five purposes in their mentoring.

#### 3.1.2 Distinct-low-focuses-on-certain-purpose clusters

##### 3.1.2.1 Cluster 1: low academics purpose mentors

Mentors in this cluster displayed a high focus on fun, sharing, character development, and future outlook purposes. However, mentors in this cluster also reported the lowest mean ratings on the *Academics purpose* (*M* = 3.65; Mean difference I-J: 1&2, −1.21, *p* < 0.01; 1&3, −1.13, *p* < 0.01; 1&4, −1.18, *p* < 0.01) when compared to the level of this purpose given by mentors in other clusters.

##### 3.1.2.2 Cluster 2: low character development purpose mentors

Mentors in this cluster rated high importance on fun, sharing, future outlook, and academics purposes. Meanwhile, they reported the lowest mean ratings on the *Character Development purpose* (*M* = 3.91; Mean difference I-J: 2&1, −0.65, *p* < 0.01; 2&3, −0.91, *p* < 0.01; 2&4, −1.26, *p* < 0.01) when compared with the other clusters.

##### 3.1.2.3 Cluster 3: low future outlook purpose mentors

Mentors in this cluster demonstrated a high focus on fun, sharing, character development, and academics purposes. On the contrary, they also reported the lowest mean ratings on the *Future Outlook purpose* (*M* = 4.02; Mean difference I-J: 3&1, −0.55, *p* < 0.01; 3&2, −0.58, *p* < 0.05; 3&4, −1.00, *p* < 0.01) when compared to the other clusters.

### 3.2 Correlations in mentor focus and mentor-mentee relationship quality by cluster

Next, we did not find significant differences among clusters on internal relational quality, internal instrumental quality nor internal match quality [Internal relational quality *F* (3, 236) = 0.559, *p* = 0.642; Internal instrumental quality *F* (3, 236) = 0.990, *p* = 0.398; Internal match quality *F* (3, 236) = 0.610, *p* = 0.609]. However, through correlation analysis within each cluster, we observed that a certain purpose on which mentors focus less within a cluster of mentors limits the improvement they can make on the mentor-mentee match quality through their other purposes.

Table 3 shows Pearson correlations of mentor purposes and mentor-mentee internal relationship quality by clusters, demonstrating statistically significant inter-cluster differences.

**Table 3:** Correlation coefficients between the mentor’s different purposes in interactions and match quality across the clusters.

Correlation		Fun	Sharing	Character development	Future outlook	Academics
Cluster 1 (*N* = 149): Low academics purpose mentors (LAPM)	Internal relational match quality	0.392^**^	0.419^**^	0.205^*^	0.216^**^	0.298^**^
	Internal instrumental match quality	0.189^*^	0.309^**^	0.211^**^	0.254^**^	0.343^**^
	Internal match quality	0.362^**^	0.419^**^	0.220^**^	0.243^**^	0.331^**^
Cluster 2 (*N* = 28): Low character development purpose mentors (LCPM)	Internal relational match quality	−0.051	0.023	0.022	−0.003	0.045
	Internal instrumental match quality	0.123	0.152	0.173	0.114	−0.021
	Internal match quality	0.010	0.057	0.068	0.033	0.020
Cluster 3 (*N* = 13): Low future outlook purpose mentors (LOPM)	Internal relational match quality	0.467	0.692^**^	0.157	0.732^**^	0.650^*^
	Internal instrumental match quality	0.328	0.444	0.021	0.496	0.469
	Internal match quality	0.433	0.628^*^	0.117	0.673^*^	0.609^*^
Cluster 4 (*N* = 50): All rounded focus mentors (ARFM)	Internal relational match quality	0.338^*^	0.416^**^	0.362^**^	0.353^*^	0.392^**^
	Internal instrumental match quality	0.460^**^	0.559^**^	0.470^**^	0.478^**^	0.538^**^
	Internal match quality	0.402^**^	0.491^**^	0.424^**^	0.419^**^	0.468^**^

**At level 0.01 (two-tailed), the correlation was significant. *At level 0.05 (two-tailed), the correlation was significant.

In ARFM cluster (cluster 4), there were moderate significant correlations between all purposes and internal match quality (fun, *r* = 0.402, *p* < 0.01; sharing, *r* = 0.491, *p* < 0.01; character development, *r* = 0.424, *p* < 0.01; future outlook, *r* = 0.419, *p* < 0.01; academics, *r* = 0.468, *p* < 0.01) by Cohen’s (Cohen, 1988) standard. To be specific, internal relational match quality was moderately correlated with sharing (*r* = 0.416, *p* < 0.01), academics (*r* = 0.392, *p* < 0.01), fun (*r* = 0.338, *p* < 0.05), character development (*r* = 0.362, *p* < 0.01), and future outlook (*r* = 0.353, *p* < 0.05). Internal instrumental match quality was moderately correlated with fun (*r* = 0.460, *p* < 0.01), character development (*r* = 0.47, *p* < 0.01), and future outlook (*r* = 0.478, *p* < 0.01), but was relatively strongly correlated with sharing (*r* = 0.559, *p* < 0.01) and academics (*r* = 0.538, *p* < 0.01).

In LAPM cluster (cluster 1), while internal match quality was small to moderately significantly correlated with rest purposes except academics (fun, *r* = 0.362, *p* < 0.01; sharing, *r* = 419, *p* < 0.01; character development, *r* = 0.220, *p* < 0.01; future outlook, *r* = 0.243, *p* < 0.01), there was moderate correlation only between relational match quality with fun (*r* = 0.392, *p* < 0.01), and sharing (*r* = 0.419, *p* < 0.01) and instrumental match quality with sharing (*r* = 0.309, *p* < 0.01).

In LCPM cluster (cluster 2), no matter whether internal relational match quality (fun *r* = −0.051, sharing *r* = 0.023, character development *r* = 0.022, future outlook *r* = −0.003, academics *r* = 0.045; *p* > 0.05), internal instrumental match quality (fun *r* = −0.123, sharing *r* = 0.152, character development *r* = 0.173, future outlook *r* = 0.114, academics *r* = −0.021; *p* > 0.05) or internal match quality (fun *r* = −0.010, sharing *r* = 0.057, character development *r* = 0.068, future outlook *r* = 0.033, academics *r* = 0.020; *p* > 0.05) were not significant correlations with any of the purposes.

In LOPM cluster (cluster 3), only internal relational match quality had a significant correlation with sharing (*r* = 0.692, *p* < 0.01) and academics (*r* = 0.65, *p* < 0.05). Internal instrumental match quality was not correlated with any other purpose (*p* > 0.05). Therefore, on the whole, there was no significant correlation between internal match quality and fun (*r* = 0.433, *p* > 0.05), character development (*r* = 0.117, *p* > 0.05), though internal match quality had significantly correlated with sharing (*r* = 0.628, *p* < 0.05) and academics (*r* = 0.609, *p* < 0.05). Results for this cluster are compromised, however, by the small sample size (*N* = 13).

Intra-cluster differences were analyzed based on the correlation analysis results. First, in LAPM where mentors place less emphasis on mentees’ academics in practice, mentors’ greater focus on other purposes (fun, sharing, character development, and future outlook purposes) still results in the increase of mentor-mentee internal relational and instrumental match quality. Second, in LOPM where mentors do not focus much on the future outlook of mentees, relational match quality can also be improved by more interactions for fun and sharing purposes, while the instrumental match quality cannot be improved by any other purpose (though again the sample size of 13 compromises these results). Finally, in LCPM where mentors do not attach much attention to character development among mentees, higher levels of interactions for fun, sharing, future outlook, and academics purposes in mentoring do not result in an increase in their relational match quality, nor instrumental match quality.

### 3.3 Demographic characteristics by clusters

For the third research question, we examined if demographic differences between mentor clusters were related to the aforementioned results. Based on Chi Square tests of independence and ANOVAs with follow-up LSD multiple comparison tests, there are no significant demographic differences among the clusters. Descriptive statistics for demographic characteristics are listed by cluster.

**Figure 3:**
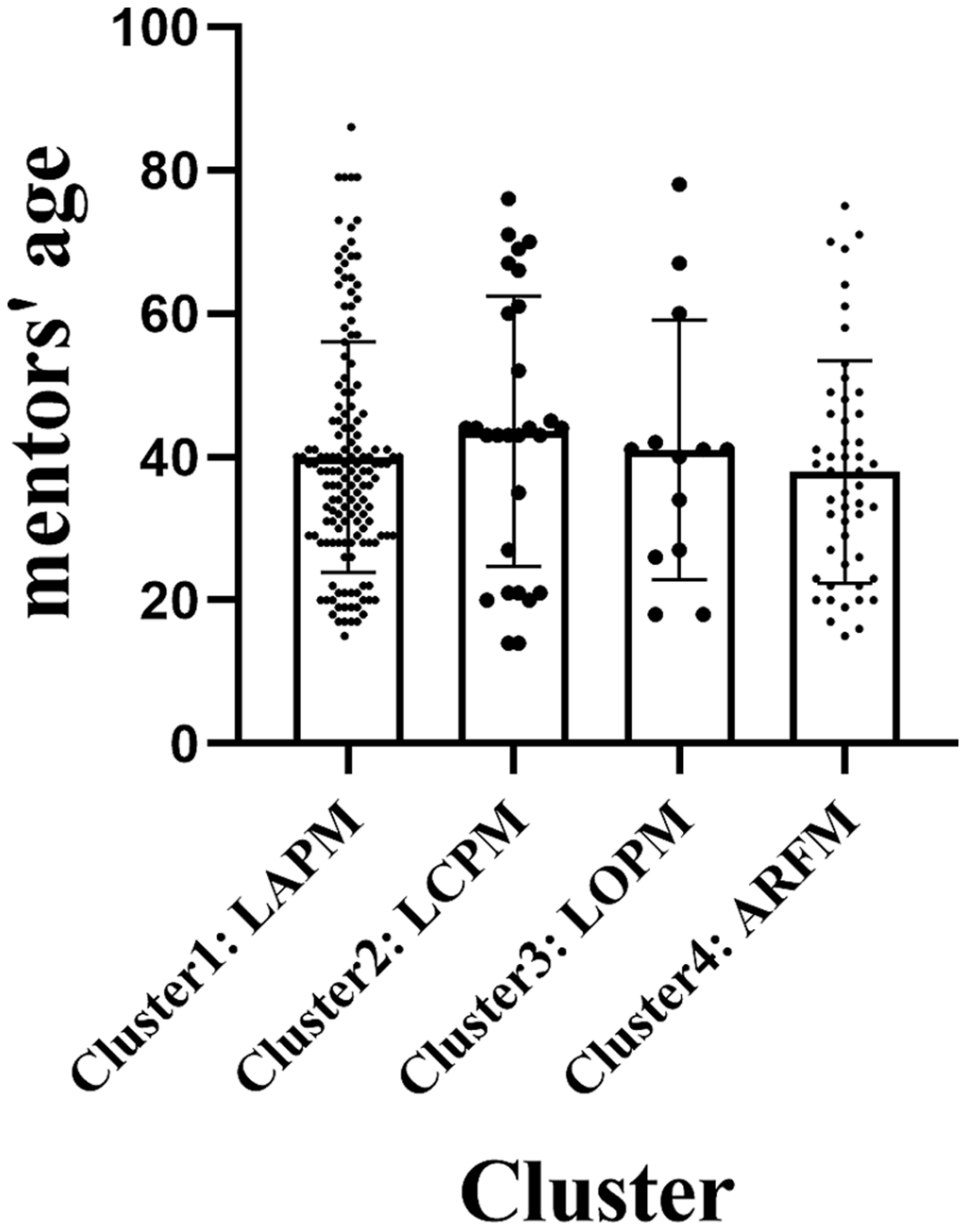
Mentors’ age descriptive statistical results.

A chi-square test for independence found no significant differences among clusters by mentors’ race (*χ*^2^ = 14.730, *p* = 0.26 > 0.05, Cramers V = 0.14) and mentors’ education background (*χ*^2^ = 20.429, *p* = 0.16 > 0.05, Cramers V = 0.17). Tables 4, 5 show the descriptive statistical results of each cluster. ANOVAs with follow-up LSD multiple comparison tests were used to check whether there were differences between clusters in mentors’ gender and age. For mentors’ age, it found that clusters had no significant differences [*F* (3, 236) = 0.73, *p* = 0.535 > 0.05]. For mentors’ gender, it found that clusters were significantly different [*F* (3, 236) = 3.175, *p* = 0.025 < 0.05]. Descriptive statistical results between clusters are reported in Figure 3 and Table 6. However, according to the results of LSD, only LAPM, in which there are fewer females, stands out as the major cluster with this significant gender difference (Mean difference I-J: LAPM&LOPM, −0.31, *p* < 0.05; LAPM&ARFM, −0.18, *p* < 0.05). The above major findings are generated by the comparison of LOPM with ARFM, LCPM with ARFM. These two sets do not have significant differences in mentors’ gender results reported by LSD (Mean difference I-J: LCPM&ARFM, −0.05, *p* = 0.649 > 0.05; LOPM&ARFM, −0.14, *p* = 0.363 > 0.05). Therefore, in the two clusters of LCPM and LOPM representing mentors’ lower attention to character development and future outlook purpose plays a crucial role in match quality, no significant gender difference were identified.

**Table 4:** Mentors’ race descriptive statistical results.

Cluster	Factor: mentors’ race	Percentage
Cluster1: LAPM	Asian	6.04%
	Black	17.45%
	Hispanic	6.71%
	White	46.31%
	Other	23.49%
Cluster2: LCPM	Asian	10.71%
	Black	10.71%
	Hispanic	3.57%
	White	46.43%
	Other	28.57%
Cluster3: LOPM	Asian	15.38%
	Black	46.15%
	White	38.46%
Cluster4: ARFM	Asian	8.00%
	Black	20.00%
	Hispanic	4.00%
	White	52.00%
	Other	16.00%

**Table 5:** Mentors’ education background descriptive statistical results.

Cluster	Factor: mentors’ education background	Percentage
Cluster1: LAPM	Some high school	11.41%
	Graduated high school	3.36%
	Some college	7.38%
	Associate’s degree or vocational certificate	19.46%
	Bachelor’s degree	32.21%
	Graduate degree	26.17%
Cluster2: LCPM	Some high school	21.43%
	Graduated high school	3.57%
	Some college	10.71%
	Associate’s degree or vocational certificate	7.14%
	Bachelor’s degree	32.14%
	Graduate degree	25.00%
Cluster3: LOPM	Some high school	23.08%
	Associate’s degree or vocational certificate	15.38%
	Bachelor’s degree	30.77%
	Graduate degree	30.77%
Cluster4: ARFM	Some high school	6.00%
	Some college	6.00%
	Associate’s degree or vocational certificate	4.00%
	Bachelor’s degree	50.00%
	Graduate degree	34.00%

**Table 6:** Mentors’ gender descriptive statistical results.

Factor: mentors’ gender (Female)	Number	Percentage
Cluster1: LAPM	45	30.20%
Cluster2: LCPM	12	42.86%
Cluster3: LOPM	8	61.54%
Cluster4: ARFM	24	48%

## 4 Discussion

Mentors, as those who take the main responsibility for relational connections in mentoring programs (Zand et al., 2009; Doty et al., 2019), often have a clear purpose of providing support to youth, such as building relationships, introducing opportunities, and developing competencies (Keller, 2005). Mentors tend to give different types of support to their mentees depending on the purpose they value. Mentor support as an important aspect of relationship quality is associated with positive adolescent development (Freedman, 1988; DuBois and Neville, 1997; Grossman and Johnson, 1999). In this study, we clustered mentors according to the importance they place on five different purposes (fun, sharing, character development, future outlook, and academics) in mentoring and examined the best results between clusters of mentors who value different purposes and mentor-mentee internal match quality, including internal relational quality and internal instrumental quality. This study found that clusters do exist among mentors with different purposes in mentor-mentee interactions. Meanwhile, there are distinct characteristics of mentor-mentee internal relationship qualities among the clusters.

### 4.1 The importance of character development focus on building mentor-mentee internal quality

In the LCPM cluster (cluster 2), mentors emphasized the importance of fun, sharing, future outlook, and academics purposes, while they rated the focus on character development comparatively lower than mentors in other clusters. This study found that mentors’ emphasis on character development can play a crucial role in mentor-mentee match quality. Mentors’ lower attention to character development may be associated with the tendency to place a greater focus on fun, sharing, future outlook, and academics purposes in mentoring. Our findings suggest that a minimal focus on character development may reduce the impact of these other purposes on both relational and instrumental match quality.

To some extent, this finding echo a series of previous research. Shaping mentees’ character is widely described as promoting the development of adolescents through the cultivation of virtues, moral values and moral agency (Pattaro, 2016). Cultivating and shaping character is often an activity advocated by schools and involving multiple parties such as family, school, and society, mainly to cultivate students’ psychological characteristics and values and shape their behavior (Lockwood, 1997). Mentors who serve as models who scaffold moral development can support the shaping of young people’s character and enhance their level of social responsibility. This is an important part of the social support that mentors can provide.

The mentors’ emphasis on supporting youth character development, as well as addressing core moral values, is conducive to helping adolescents construct their “personal meaning” (Selman, 2003) and development of high-quality relationships. When interpreting his theory of interpersonal development, Selman (2003) elaborated on “personal meaning” as a social thinking and cognition, which is a reflection of an individual’s self-understanding and personal values. Adolescents come to make meaning of and value (or devalue) their interpersonal possibilities based on what they have experienced throughout their relational histories. In this way, adolescents begin to construct personal meaning and achieve social and self-development. Meanwhile, mentors who value character development purpose, often guide by emphasizing the characteristics of care, mutual respect, and fairness, in order to inculcate the content and essence of values education and stimulate a network of systemic “relational trust” (Bryk and Schneider, 1996, 2002), which in turn can enhance mentoring relationship quality and positive outcomes for mentees (Nakkula and Harris, 2005). Mentors can play a role in the process of acquiring social experiences for older children and adolescents by providing scaffolds for character development, affecting their understanding of how they see themselves and others, and building a code for constructive moral values together. When mentors and mentees explore and discuss shared moral values, the relationship between them may be more stable and meaningful. If the mentor and mentee do not form a meaningful relationship, the mentors’ focus on other aspects such as fun, sharing, academics, etc. and future outlook may be less effective in promoting the match relationship quality. In summary, our findings suggest that mentors who do not focus adequately on character development risk reducing the personal meaning of the relationship for their mentees and, in turn, overall match quality.

### 4.2 The significance of future outlook guidance on building internal instrumental quality

In the LOPM cluster (cluster 3), mentors displayed significant emphasis on fun, sharing, character development, and academics purposes. However, in contrast, they provided relatively lower ratings for future outlook purpose. Another finding in this study is that future outlook purpose plays a relatively important role in instrumental match quality. During the actual mentoring process, in the absence of mentors’ attention to future outlook, interactions centered around fun and sharing purposes can still positively influence relational match quality. On the other hand, instrumental match quality was unaffected by any specific purpose when the emphasis on future outlook was relatively low.

The future outlook focus represents the mentors’ emphasis on understanding the life purpose of the mentees, as well as the responsibility to assist them in exploring the directions of their life and considering general plans for pursuing life interests. Studies have revealed that youth with a clear life purpose reported having close, long-term relationships with mentors who helped them discover and pursue their purposes (Bronk, 2012). Teachers who show genuine interest and concern for their students’ life ideals can foster stronger teacher-student relationships. When teachers actively listen to students’ aspirations and provide support and guidance, students feel valued and understood. This kind of concern goes beyond academic performance and focuses on students’ personal development and future goals. Through this connection, trust, and emotional bonds are established, resulting in a positive teacher-student relationship (Nakkula and Harris, 2005; Bronk, 2012). The teacher’s attention to students’ life goals can help develop their self-awareness and goal-setting abilities, stimulate their creativity, innovative thinking, self-reflection, and growth awareness, and promote students’ emotional and psychological well-being (Burrow et al., 2010); and cultivate their sense of social responsibility and civic consciousness (Parks, 2011; Bronk, 2012). All this personalized attention and guidance can not only promote mutual inspiration and positive influence between teachers and students, but also enhance the connection positive interactions between them, which is central to establishing a positive teacher-student relationship.

In this sense, when mentors understand the youth’s developmental needs and help them explore their life goals, they may be more capable of offering support that is meaningful for the youth’s specific needs and goals. In the meantime, when becoming clearer on their life purposes, mentees will be better positioned to actively seek support from their mentors. This specific focus on the mentee’s life purpose from both sides may result in a high-quality instrumental mentor-mentee relationship. This research testifies to such an opinion from the opposite side that if mentors have less focus on future outlook and do not attempt to understand the youth’s life goals, they may not be able to provide targeted resources and support based on the youth’s developmental plan. And when mentors place the least focus on mentees’ life purpose and future directions and do not exert much time and energy to understand and help mentees find their purpose in life, their other purposes in their interactions with mentees, such as fun, sharing, academics, and character development may be less effective in promoting higher mentor-mentee instrumental match quality.

### 4.3 Cultivating mentors with the focus on both mentors’ and mentees’ character and future outlook

In order to promote a better mentor-mentee match quality, our research suggests mentors endow ample focus on mentees’ character and life goal development. This not only requires mentors to impart sufficient attention to the formation and development of youngsters’ character traits and future outlook, using appropriate techniques and approaches, but also the cultivation and development of their own character traits and life purposes as prerequisites.

This stance requires mentors to have the appropriate competencies and character to help develop the youth’s character, help them establish life goals and form their self-identity. First, the character, beliefs, and values of the mentors themselves are critical to the successful implementation of character education as an important aspect of mentoring. Therefore, as with teachers, raising mentors’ willingness to engage in character education should be an integral part of training and development programs (Berkowitz and Bier, 2007). Mentors should be aware of their personal meaning regarding morally related issues discussed in mentoring sessions given that they serve, in part, as moral role models (Jackson et al., 1993; Campbell, 2000; Fenstermacher, 2001). In this sense, in mentor education processes, mentors should be made aware of the importance of mentors’ focus on mentees’ character development and future outlook on promoting a higher mentor-mentee internal match quality, which could ultimately lead to more positive outcomes of youth. Only if mentors understand their own personal meaning and establish lasting and prosocial moral standards, can they create a scaffold regarding positive character development and outlook for youth in their actual mentoring activities.

Meanwhile, teachers who have a comprehensive understanding of their students’ individual needs and characteristics tend to develop more positive teacher-student relationships, because through understanding their students, teachers can better respond to their needs, foster interaction, and establish a positive emotional connection (Roorda et al., 2011). Similarly, mentors must strive to understand their mentees by committing to an approach that seeks insights from multiple sources about what might be going on for them (Nakkula and Toshalis, 2006). Young people learn the meaning of life and develop such meanings as they continue to receive life experiences. The premise here is that mentors play a key role in the process of youth gaining life experience as they adopt an orientation toward their mentees that suggests a posture of humility, advocacy, and ongoing learning about how young people see things and what they may need to grow. In this way, the mentor provides an appropriate scaffold for young people, which not only meets their needs to explore the world, but also stays within the scope of youth self-realization, thus helping them establish strong character, clear life goals, and a deeper understanding of the meaning of their lives.

Finally, finding an appropriate blend of mentoring strategies is essential. Warmth, responsiveness, and supportive communication foster trust, engagement, and a sense of connection between teachers and students (Pianta et al., 2003). Positive teacher-student interaction methods contribute to improving the quality of teacher-student relationships and help to establish connectedness and a sense of community (Roorda et al., 2011), foster ethical decision-making skills (Nucci and Narvaez, 2008), develop moral reasoning, practice ethical behavior, and reflect on their values and life ideals (Lickona, 1991). Similarly, the mentor’s approach, or what the mentor does to build the relationship (e.g., activities chosen, discussion topics, developmental opportunities), is related to how mentors can support youth and therefore what kind of impact they provide (Parra et al., 2002; Spencer, 2012). When the mentor’s approach fits with the specific situation of the match (e.g., personalities, communication preferences, youth’s developmental stage), youth are better able to utilize scaffolding from the mentor to achieve better developmental outcomes.

### 4.4 Limitations

We have mentioned a number of points that limit the conclusions of our findings, including the small size of the sample and reliance on subjective results reported by mentors.

The major limitations of our findings are due to the small size of the sample and the limited nature of the relationships represented. The results we have obtained may be more accurate and representative with a larger sample. In addition, this study relied on reports from mentors of how they supported the youth and match quality perceived by the mentors, not objective measures of change. It is possible that participants who are satisfied with their relationships provide more support due to positive feelings about the relationship, while mentors who feel unsatisfied with the relationship or fail to recognize positive effects of the relationship will provide less support. To this end, we recommend that future studies also use intensive observational measures, such as those used by Keller and Pryce (2012), Pryce (2012), and Pryce and Keller (2011), in order to objectively measure interactions, conversations and attempts at mentor support.

Additionally, the scope of our research was limited to in-person mentoring relationships. Future research could be extended to virtual mentoring which is now widely practiced. For example, in the context of the metaverse, teacher-student relationships exhibit different characteristics in terms of the knowledge foundation, role identity, practice methods and value norms, shaping a new paradigm of teacher-student relationships (Feng, 2022).

## 5 Conclusion

In the field of youth mentoring, mentor support is an important scaffold for youth development. According to the current study, there is a link between the support provided by different categories of mentors and match relationship quality. Specifically, mentors with a low focus on mentees’ character development and future outlook appear to hinder the impact of their other forms of interactions such as fun, sharing, or academics in promoting high mentor-mentee match relationship quality, which may serve as an important indicator of mentoring effectiveness, leading to mentees’ improved developmental outcomes. Therefore, policymakers, researchers, and mentors should give particular consideration to the focus in mentor-mentee interactions on character development and future outlook in program in program design and mentoring approaches.

## Data availability statement

The original contributions presented in the study are included in the article/supplementary materials, further inquiries can be directed to the corresponding author.

## Ethics statement

The studies involving human participants were reviewed and approved for secondary data analysis by the University of Pennsylvania Internal Review Board. Participants were informed during the data collection stage that the data collected with the surveys, including demographic data and programmatic descriptors, would be used to improve the instruments and further study for secondary data analysis by the University of Pennsylvania Internal Review Board.

## Author contributions

BF: Writing – original draft, Writing – review & editing. MN: Writing – review & editing. FJ: Writing – review & editing.
